# Peroxisome proliferator-activated receptor agonist rosiglitazone improves host defense against *Pseudomonas aeruginosa *in a murine model of pneumonia

**DOI:** 10.1186/cc13010

**Published:** 2013-11-05

**Authors:** Crisitina Lyra Nagae, Cassiano Felippe Gonçalves-de-Albuquerque, Alessandra Silveira Ferreira, Raysa Captivo, Larissa Camisão, Carlos André Mandarino Silva, Mirian Priscila Lins de Lima, Caroline Loureiro Hildebrandt, Patricia Torres Bozza, Hugo Caire de Castro-Faria-Neto, Adriana Ribeiro Silva

**Affiliations:** 1 Laboratório de Imunofarmacologia, IOC/FIOCRUZ, Rio de Janeiro, RJ, Brazil

## Background

*Pseudomonas aeruginosa *is a Gram-negative bacterium regarded as an opportunistic pathogen. It infects immunocompromised patients, and is the second leading cause of nosocomial diseases. This bacterium has numerous virulence factors, adapts quickly to new environments, and requires a few nutrients to survive. All of these mechanisms will generate a host response. The fastest immune response is neutrophil recruitment, followed by phagocytosis and degranulation. There is another mechanism to fight bacteria called NET formation, which is the formation of a neutrophil extracellular network. NET is formed through a process called NETosis where the release of the cell nuclear material can hold and destroy pathogens. The nuclear receptor peroxisome proliferator-activated receptor PPARγ, besides lipid and glucose metabolism, is involved in the inflammatory response modulation, being considered a potential target for the study of new therapies for inflammatory and infectious diseases. We therefore aim to investigate the involvement of PPARγ in lung injury caused by *P. aeruginosa *using an agonist of this receptor, rosiglitazone.

## Materials and methods

For this purpose, Swiss mice were instilled intratracheally with bacteria and treated with rosiglitazone 5 hours after the operation. We analysed clinical signs using 10 physical parameters, cellularity and DNA measurement to assess NET formation.

## Results

We found that the animals stimulated with *Pseudomonas *showed an increase in inflammatory parameters, while the animals treated with rosiglitazone showed improvement in clinical signs and increased NET formation.

## Conclusions

We can conclude that rosiglitazone has an anti-inflammatory role during lung infection, suggesting that PPARγ activation may improve the host defense against bacteria.

